# Prognostic value of serum macrophage migration inhibitory factor levels in pulmonary tuberculosis

**DOI:** 10.1186/s12931-019-1004-3

**Published:** 2019-03-06

**Authors:** Qingjiang Wang, Wei Han, Junmei Niu, Bing Sun, Wei Dong, Guangpeng Li

**Affiliations:** Department of Tuberculosis, The first affiliated Hospital of Xinxiang Medical University, No.88, Jiankang road, Weihui, Xinxiang, 453100 China

**Keywords:** Macrophage migration inhibitory factor, Prognosis, pulmonary tuberculosis, Chinese

## Abstract

**Background:**

Macrophage migration inhibitory factor (MIF) makes chemokine-like functions and plays critical roles in various inflammatory diseases. This study was designed to explore the significance of MIF serum levels in predicting the prognosis of pulmonary tuberculosis (PTB) following anti-TB treatment.

**Methods:**

Patients diagnosed with culture-confirmed PTB without treatment were included and the serum was collected. Levels of MIF in serum were quantified with immunoassay, and the levels of established biomarkers were also determined, including C-reactive protein (CRP) and Interleukin 6 (IL-6). The outcome was estimated with all-cause mortality, with the mortality in 12 months as the primary outcome and the mortality in 3, 6, 9 months as other outcomes. The prognostic value of MIF and other factors in PTB were tested.

**Results:**

Two hundred eighty-seven PTB patients were included. The median MIF levels in patients with advanced disease, disseminated and drug-resistant TB were significantly higher than that observed in mild -to- moderate disease, non-disseminated and drug-sensitive TB. MIF levels in patients with the outcome of death were higher than those survived [28.0 ng/ml (Inter-quartile range [IQR]: 24.2–33.1) vs. 22.3 ng/ml (IQR: 18.7–26.5); *P* < 0.001]. Multivariate model analysis was performed for comparing the highest quartiles to the lowest quartile of MIF levels. MIF levels were related to the mortality, with an elevated mortality risk of 236% [Odds ratio (OR) = 3.36; 95% Confidence interval (CI): 1.21–15.14; *P* = 0.012]. The model was re-analysis after combing MIF with currently established risk indicators. The obtained Area Under the Receiver Operating Characteristic Curve (±standard error) was elevated from 0.81 (±0.035) to 0.84 (±0.031), with a significant difference before and after adding the MIF (difference, 0.03[0.004]; *P* = 0.03).

**Conclusion:**

Serum level of MIF was a better biomarker than CRP or IL-6 for predicting death in HIV-negative PTB patients, and increased MIF serum levels were related to higher mortality.

## Introduction

*Mycobacterium tuberculosis* (MTB) is an infectious-agent for tuberculosis (TB), which has been leading cause of death for humans. Until now, TB has still given rise to a heavy public health burden in developing countries [1]. The crude (age-adjusted) incidence was annually decreased by 4.2% (5.1%); while the crude (age-adjusted) mortality was annually decreased by 9.7% (12·4%) [2]. A tuberculosis control program was scaled up in China [3]. The incidence of smear-positive tuberculosis was decreased, from 170 cases per 100, 000 population in 1990 to 59 cases in 2010 [4]. However, the prevalence of drug-resistant tuberculosis was severe in China [5]. Therefore, the TB control and prevention is still challenging, especially the early diagnosis and effective therapy for drug-resistant tuberculosis.

For pulmonary TB (PTB), the cellular immune response toward the MTB has been the character. The macrophages and lymphocytes would be activated with MTB, and then the cytokines would be secreted, including tumor necrosis factor-α (TNF-α), Interleukin (IL)-6, IL-8, and IL-12 [6]. Some studies have reported the application of some other cytokines as biomarkers, including the C-Reactive Protein (CRP) and IL-2 [7, 8]. The levels of these biomarkers were related to the disease severity [7, 8]. In a previous study, the levels of red cell distribution width (RDW) and neutrophil to lymphocyte ratio (NLR) were applied for indicating inflammation in TB patients, as well as managing disease progression [9].

Macrophage migration inhibitory factor (MIF) makes chemokine-like functions and plays critical roles in several acute and chronic inflammatory diseases. [10, 11]. MIF has also played roles in immuno-regulation and initiate an innate immune response. It proposed that MIF could induce the production of TNF-α and other pro-inflammatory cytokines [12, 13]. Furthermore, serum levels of MIF were reported to be a useful marker for assessing the effects of therapy in active pulmonary tuberculosis (APTB) patients [14]. Another study suggested a distinct genetic and immunopathogenic basis for TB at the MIF locus and serum MIF, IFN-γ and TNF-α profiles distinguish TB from the more inflammatory phenotype, as well as making effects in pathogenesis and as biomarkers of active TB [15]. Until now, there was few studies exploring the application of MIF as a biomarker for predicting inflammatory pathway prognosis in PTB patients. This study was designed to explore the correlation between serum levels of MIF in PTB patients and the disease prognosis following anti-TB treatment.

## Patients and methods

### Study population

From January 2014 to December 2016, the study was performed in the first affiliated Hospital of Xinxiang Medical University. The patients(≥18 years) diagnosed with culture-confirmed PTB and without treatment were prospectively recruited. Those patients were defined as the training cohort. At the same time, the validation cohort recruited 104 consecutive PTB patients without treatment from Weihui People’s Hospital. The PTB had been diagnosed per the Diagnostic Standards and Classification of Tuberculosis (the 1990 edition) [16]. Culture confirmed PTB were isolated MTB from sputum and new infiltrates in pulmonary radiography [17]. Cases with the following conditions were excluded: (1) HIV or other pathogens infection other than MTB; (2) malignant tumor; (3) receiving operation or trauma during the past 3 months; (4) liver and kidney function insufficiency; (5) rheumatoid arthritis, pneumonia, insufficient immuno-function that susceptible to opportunistic infection and other respiratory diseases. The included patients had not received anticoagulant therapy, nonsteroidal anti-inflammatory drugs, or contraceptives.

In this study, 100 community-acquired pneumonia (CAP) patients were involved as control group 1, with matched age and sex. The CAP was pneumonia initiated at home without applying antibiotics in previous 2 weeks. The CAP was diagnosed per following standards: respiratory symptoms such as cough and sputum; inflammatory symptoms such as fever and increased CRP level; no acid-fast bacteria was observed; no abnormal chest X-ray shadows was presented. For the control group 2, 100 age and sex matched healthy controls were enrolled from the same population. The healthy controls were primarily identified with self-report and then presented with negative results in T-SPOT.TB assays. The healthy controls were also performed routine chest X-ray examination to rule out possible lung abnormalities. The study was approved by the ethics committee of First Affiliated Hospital of Xinxiang Medical University. The informed consent was interpreted and obtained from all included patients.

### Clinical information

Following data were collected: sex, age, body mass index (BMI), smoking or not, excessive drinking, previous TB history, comorbidities, blood routines, microbiologic culture and radiography. In addition, the sputum of all the patients was characterized with aci-fast smears and mycobacterial culture. A standard 4-drug regimen was administrated for all included PTB patients: daily isoniazid (H), rifampicin (R), ethambutol (E), and pyrazinamide (Z) in the intensive phase of first 2-month and HRE in the following continuation phase. The disseminated TB was defined as TB infection at more than 2 other non-contiguous organs [17, 18]. The cavitary status and whether unilateral or bilateral involvement was determined with chest radiography. The disease was graded as minimal, moderate and advanced disease based on imaging results per the classification of the National Tuberculosis and Respiratory Disease Association [19]. The minimal disease only involved single-lobe, without pleural involvement or visible cavity; moderate disease involved two or more lobes, with mild pleural involvement or effusion, or a solitary cavity; and advance disease referred to bilateral involvement, moderate pleural involvement or effusion, and multiple cavities. The disagreement was determined with consensus. The severity of both PTB and CAP patients was evaluated with previously reported scale [20]. The outcome was estimated with all-cause mortality, with the mortality in 12 months as the primary outcome and the mortality in 3, 6, 9 months as other outcomes. The follow-up of all the patients were lasted for 12 months after the diagnosis of PTB, and it would be discontinued if patient died or loss of contact.

### Samples and assays

For all the included patients, the blood was sampled via venipuncture at 6:00 am after the admission during fasting. For some patients, the blood was collected at five time-points: at admission (before any treatment), 3, 6, 9 and 12 months after standard anti-TB treatment. The whole blood was centrifuged under 1000×g for 12 min for obtaining serum. All the obtained samples were preserved at − 80 °C pending assay. The serum was diluted to 10 times and the MIF levels were tested with a commercially Quantikine Human MIF ELISA kit (DMF00B, R&D, Minneapolis, USA). The range of measurement for MIF was 0.2–10 ng/ml. The intra- and inter-assay reproducibility was characterized with coefficients of variation (CV), which was 4.0–6.5% and 6.5–8.0%, respectively. IL-6 levels in serum were also quantified with ELISA. In addition, blood routine examination of all the blood samples were performed with standard laboratory methods, for obtaining the blood levels of CRP, white blood cell count (WBC), fasting blood glucose (FBG) and albumin (ALB).

### Statistics

The data of categorical variable was expressed as percentage and the data of continuous variable was expressed as median (quartile). Both the chi-square test and Mann–Whitney U-test were applied for comparing proportions for the asymmetrically distributed variable. Spearman’s Rank correlation coefficient was applied for evaluating the bivariate correlation.

The univariate and multivariate binary logistic regression analysis was applied for exploring the predicting significance of MIF on disease progression, dissemination, drug resistance and mortality. The confounding factors were obtained with the univariate analysis and included age, cavitary lesion, pleural effusion, drug-resistant, disseminated, ALB, CRP, WBC, IL-6 and MIF. After adjusting for these confounding factors, the odds ratio (OR) with corresponding 95% Confidence interval (CI) was calculated. In addition, multivariate analysis was also applied for calculating the adjusted OR with 95% CI, which was performed to estimate the association between MIF quartiles and mortality (with the reference of the lowest MIF quartile).

Second, the receiver operating characteristic (ROC) curve was performed for determining the cut-off value, which was the serum MIF levels at admission for predicting outcomes with the highest sensitivity and specificity. The Area under the curve (AUC) was also obtained for characterizing the accuracy. The indicators of Integrated discrimination improvement (IDI) and net reclassification improvement (NRI) were obtained to evaluating the clinical availability after adding MIF to established risk indicators, as well as the improvement of MIF on mortality prediction [21].

Lastly, the Kaplan–Meier analysis and log-rank test was performed for analyzing the association between quartiles of serum MIF levels and mortality within 12-month of admission. SPSS (version 22.0, SPSS Inc., Chicago, IL, USA) was applied for performing all statistics. *P* < 0.05 was considered as significant in statistics.

## Results

### Baseline clinical characteristics of the patients in the training cohort

Blood was available for 287 patients with PTB. The median serum level of MIF was 23.0 ng/ml (IQR, 19.1–28.1) and it was significantly lower compared to that of in CAP patients, which was 27.7 ng/ml (IQR: 21.7–33.5), but it was higher than that of in HC controls (14.3; IQR: 10.9–17.2) ng/ml. The median age at admission was 57 (IQR, 49–63) years old, and 149(51.9%) were man. As shown in the Table 1, there were 165 (57.5%) cases of sputum acid-fast smear-positive, 106 (36.9%) cases of cavity and 54 (18.8%) cases of pleural effusion. From the radiographic results, the minimal to mild disease took account for 55.7% of cases, while the advanced disease accounted for only 19.2%.

**Table 1:** Patients characteristics at admission

Characteristics	PTB	CAP	Control
N	287	100	100
Age (years), median (IQR)	57 (49–63)	57 (48–63)	57 (48–63)
Sex-male, n (%)	149 (51.9)	52 (52.0)	52 (52.0)
BMI (kg/m2), median (IQR)	24.3 (23.1–25.9)^†‡^	25.8 (24.3–27.4)	26.0 (24.8–28.5)
Current smoker, n (%)	63 (22.0)	24 (24.0)	21 (21.0)
Alcoholism, n (%)	13 (4.5)	4 (4.0)	3 (3.0)
Past history of tuberculosis, n (%)	19 (6.6)	–	–
Symptoms, n (%)			
Fever	133 (46.3)^†^	100 (100.0)	–
Cough or sputum	148 (51.6)^†^	69 (69.0)	–
Dyspnoea	55 (19.2)^†^	59 (59.0)	–
Chest pain	21 (7.3)	5 (5.0)	–
Wight loss	53 (18.5)^†^	3 (3.0)	–
Radiography findings			
Cavitary lesion	106 (36.9)^†^	13 (13.0)	–
Pleural effusion	54 (18.8)	14 (14.0)	–
PORT score	90.5 (80.7–105.6)^†^	104.8 (92.5–116.3)	–
Positive sputum acid-fast smear, n (%)	165 (57.5)	–	–
Extent of disease, n (%)			
Minimal to mild disease	160 (55.7)	–	–
Moderate	72 (25.1)	–	–
Advanced disease	55 (19.2)	–	–
Drug resistant tuberculosisa, n (%)	38 (13.2)	–	–
Disseminated tuberculosis, n (%)	31 (10.8)	–	–
Death, n (%)	36 (12.5)	3 (3.0)	–
Laboratory test			
WBC 10^9^/L	7.95 (7.10–9.33)^†‡^	12.1 (9.7–14.2)	5.88 (5.02–7.13)
CRP, mg/dl	5.14 (4.03–6.55)^†‡^	10.15 (6.12–13.21)	0.23 (0.13–0.52)
IL-6, ng/ml	10.8 (9.0–13.2)^†‡^	12.5 (9.8–16.6)	9.4 (6.9–11.9)
MIF, ng/ml	23.0 (19.1–28.1)^†‡^	27.7 (21.7–33.5)	14.3 (10.9–17.2)
FBG, mmol/l	5.79 (5.01–6.21)	5.66 (4.93–6.02)	5.38 (4.89–5.76)
Albumin, g/dl	3.4 (3.2–3.7)	3.4 (3.1–3.7)	3.5 (3.2–3.7)

Data are median (interquartile range) or n (%)

†compared with CAP groups by chi-square or Mann–Whitney test and *P* < 0.05

‡compared with HC groups by chi-square or Mann–Whitney test and *P* < 0.05

*PTB* Pulmonary Tuberculosis, *CAP* community-acquired pneumonia, *BMI* body mass index, *WBC* white blood cell count, *CRP* C-reactive protein, *FBG* fasting blood glucose, *IL-6* Interleukin 6, *MIF* macrophage migration inhibitory factor

In patients with PTB, the serum levels of MIF were modestly correlated to the levels of CRP (r = 0.172, *P* = 0.003) and IL-6 (r = 0.203, *P* = 0.001). Serum levels of MIF was correlated to neither sex (*P* = 0.193) nor age (*P* = 0.106). In addition, there was no association between MIF serum levels and others markers, including BMI, current smoker, prior history of tuberculosis, and positive sputum acid-fast smear (*P* > 0.05, respectively). Interestingly, the data suggested that patients with cavitary lesion and pleural effusion had significantly higher serum levels of MIF than that of the other patients [27.5(23.2–30.5) vs. 21.6(18.0–26.5) ng/ml, *P* < 0.001 and 26.5(22.1–29.5) vs. 21.9 (18.5–26.9) ng/ml, *P* = 0.001].

### MIF and severity of the disease in the training cohort

**Fig. 1:**
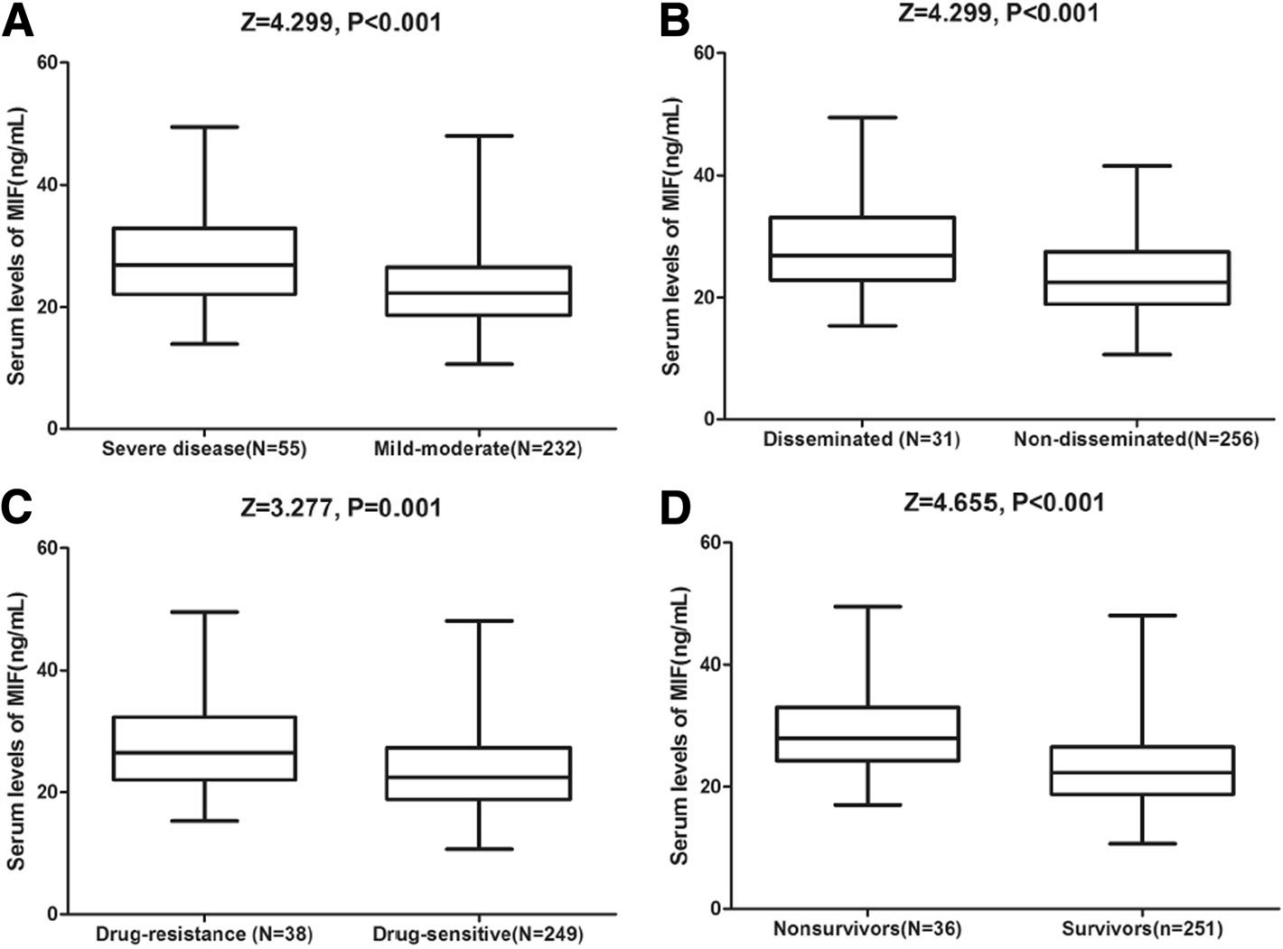
Serum levels of MIF in different groups. **a** serum levels of MIF in advanced disease and mild-to-moderate clinical severity; (**b**) serum levels of MIF in disseminated and non-disseminated PTB; (**c**) serum levels of MIF in drug-resistant and drug-sensitive PTB; (**d**) serum levels of MIF in survivors and non- survivors. All data are medians and in-terquartile ranges (IQR); *P* values refer to Mann-Whitney U tests for differences between groups(*P* < 0.0001). MIF = Macrophage migration inhibitory factor; PTB=Pulmonary Tuberculosis

At admission, 55 patients (19.2%) had an advanced disease. The median MIF levels in serum were higher in patients with advanced disease, compared to those observed in mild -to- moderate severity (26.8[22.1–32.9] vs. 22.3[IQR, 18.6–26.5] ng/ml, *P* < 0.001; Fig. 1a). The univariate and multivariate logistic regression analysis was performed. When serum level of MIF was increased by 1 ng/ml, the unadjusted risk for advanced disease would be elevated by 9% (OR: 1.09 [95% CI: 1.04–1.14], *P* < 0.001); and the adjusted (including age, cavitary lesion, pleural effusion, drug-resistant, disseminated, ALB, CRP, WBC and IL-6) risk would be elevated by 5% (OR: 1.05 [95% CI: 1.01–1.11], *P* = 0.003). The ROC curves were plotted for exploring the optimal MIF value in predicting the advanced disease. When serum level of MIF was at 28.1 ng/ml, the highest sensitivity of 49.1% and specificity of 80.2% could be obtained [AUC = 0.69, 95% CI: 0.61–0.77; *P* < 0.001]. The accuracy of MIF level for predicting advanced disease was higher than that of CRP level [AUC = 0.61, 95% CI: 0.53–0.69, *P* = 0.001], WBC [AUC = 0.58, 95% CI: 0.51–0.66, *P* < 0.001] and IL-6 [AUC = 0.62, 95% CI: 0.54–0.70, *P* = 0.003].

Disseminated TB was diagnosed in 31 (10.8%) patients. The median MIF levels in serum were higher in patients with disseminated TB, compared to those observed in non-disseminated TB (26.8[22.8–33.1] vs. 22.4[IQR, 18.9–27.4] ng/ml, *P* < 0.001; Fig. 1b). The univariate and multivariate logistic regression analysis was performed. When serum level of MIF was increased by 1 ng/ml, the unadjusted risk for disseminated TB would be increased by 8% (OR: 1.08 [95% CI: 1.03–1.14], *P* = 0.005); and the adjusted (including age, cavitary lesion, pleural effusion, drug-resistant, ALB, CRP, WBC and IL-6) risk would be elevated by 3% (OR: 1.03 [95% CI: 1.00–1.10], *P* = 0.016). The ROC curves were plotted for exploring the optimal MIF value in predicting the advanced disease. When serum level of MIF was at 23.8 ng/ml, the highest sensitivity of 74.2% and specificity of 59.0% could be obtained [AUC = 0.68, 95% CI: 0.58–0.78; *P* < 0.001]. The accuracy of MIF level for predicting disseminated TB was higher than that of CRP level [AUC = 0.63, 95% CI: 0.51–0.75), *P* = 0.036], WBC [AUC = 0.54, 95% CI: 0.43–0.65, *P* < 0.001] and IL-6 [AUC = 0.66, 95% CI: 0.57–0.76, *P* = 0.45].

Drug-resistant TB was diagnosed in 38 (13.2%) patients (Isoniazid-18 patients, Streptomycin-25, Rifampin-8, Ethambutol-6, Multidrug resistance-7). The median MIF levels in serum were higher in patients with drug-resistant TB, compared to those observed in drug-sensitive cases (26.5[22.0–32.3] vs. 22.4[IQR, 18.8–27.3] ng/ml, *P* < 0.001; Fig. 1c). The univariate and multivariate logistic regression analysis was performed. When serum level of MIF was increased by 1 ng/ml, the unadjusted risk for drug-resistant TB would be increased by 8% (OR: 1.08 [95% CI: 1.03–1.14], *P* = 0.002); and the adjusted (including age, cavitary lesion, pleural effusion, disseminated, ALB, CRP, WBC and IL-6) risk would be elevated by 4% (OR: 1.04 [95% CI: 1.01–1.09], *P* = 0.012). The ROC curves were plotted for exploring the optimal MIF value in predicting the advanced disease. When serum level of MIF was at 24.8 ng/ml, the highest sensitivity of 63.2% and specificity of 63.9% could be obtained [AUC = 0.67, 95% CI: 0.58–0.75; *P* < 0.001]. The accuracy of MIF level for predicting drug-resistant TB was higher than that of CRP level [AUC = 0.60, 95% CI: 0.53–0.69, *P* = 0.015], WBC [AUC = 0.55, 95% CI: 0.47–0.63, *P* < 0.001] and IL-6 [AUC = 0.64, 95% CI: 0.55–0.73, *P* = 0.37].

**Fig. 2:**
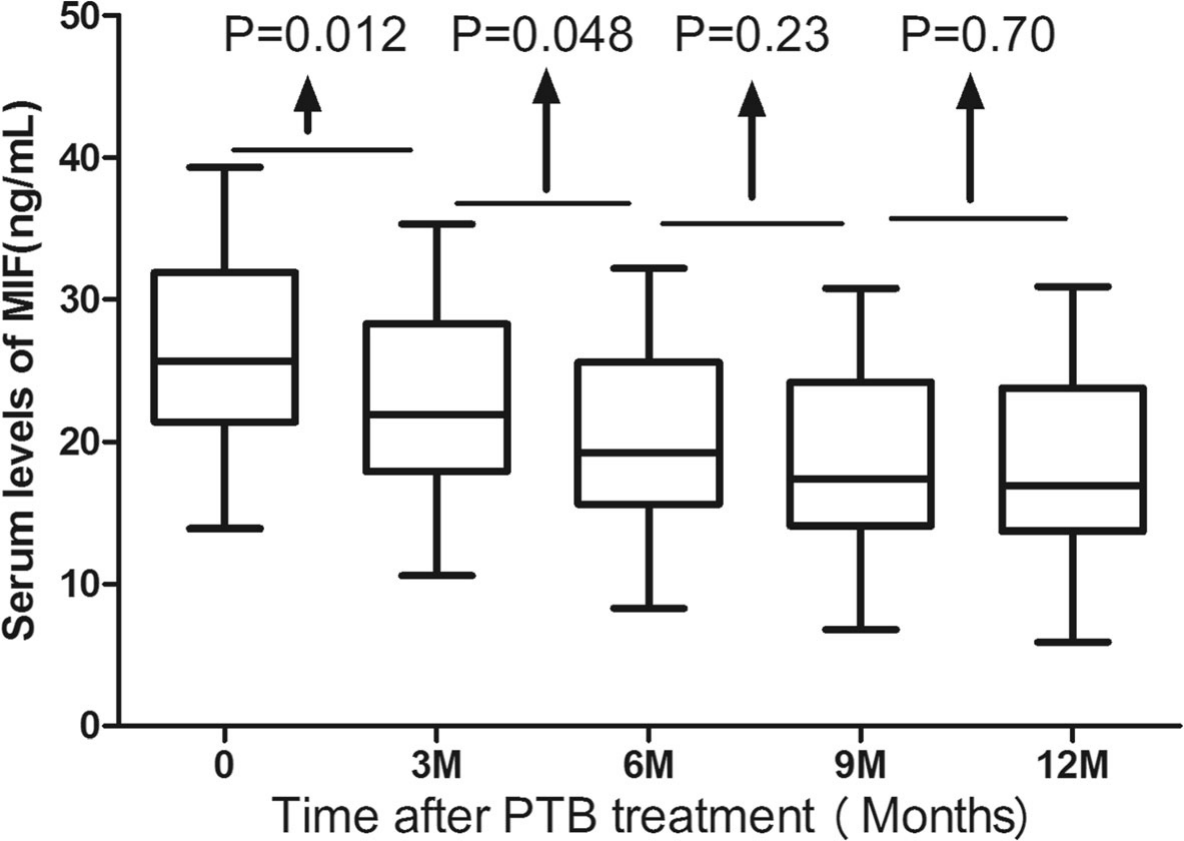
Box plots (median, interquartile ranges) of serum levels of MIF in the 5 time-point after PTB treatment (*N* = 47). MIF = Macrophage migration inhibitory factor; PTB=Pulmonary Tuberculosis

Blood samples were obtained at follow-up time points in a subgroup of 48 patients. The variation of serum MIF level with time course could be presented and there were significant variations with sampling time points (*P* < 0.001). The peak was observed on month 0 (*P* < 0.001, compared to month 3, 6, 9 and 12), and followed by a platform between month 9 to 12 (Fig. 2). Therefore, the above results suggested that the serum levels of MIF were quickly decreased from 0 month to 12th month after the treatment.

### MIF and death in the training cohort

MIF levels in patients with the outcome of death were higher than those survived [28.0 ng/ml (IQR: 24.2–33.1) vs. 22.3 ng/ml (IQR: 18.7–26.5); *P* < 0.001; Fig.1d]. Univariate analysis identified death related biomarkers, mainly including MIF levels, age, cavitary lesion, pleural effusion, drug-resistant, disseminated status and CRP, IL-6 and ALB (Table 2). After adjusting for these biomarkers, the MIF level could still independently predict mortality, with an OR of 1.06 (95% CI = 1.01–1.15; *P* = 0.009). The multivariate analysis was also performed for obtaining the adjusted OR with 95% CI for mortality of MIF quartiles (with the reference of lowest quartile). Multivariate analysis was performed and the fourth quartile was compared to the first quartile of MIF (Table 3), the MIF was associated with mortality and after adjusting, the risk of mortality would be elevated by 236% (OR: 3.36 [95% CI: 1.21–15.14], *P* = 0.012).

**Table 2:** Prediction of 12-month mortality in univariate and multivariate logistic regression analysis

Parameter	Univariate Analysis			Multivariate Analysis^ξ^		
	OR	95% CI	*P*	OR	95% CI	*P*
Age (per one unit)	1.06	1.02–1.11	0.004	1.04	1.01–1.09	0.012
Cavitary lesion	2.55	1.67–4.16	0.014	1.65	1.09–3.07	0.048
Pleural effusion	2.32	1.32–3.98	0.020	1.79	1.05–2.99	0.075
Drug-resistant	3.60	2.30–5.71	< 0.001	2.25	1.40–3.00	0.009
Disseminated	2.55	1.45–3.65	< 0.001	1.73	1.22–2.45	0.004
ALB (per one unit)	0.59	0.48–0.79	< 0.001	0.79	0.69–0.93	0.008
CRP (per one unit)	1.35	1.17–1.53	0.009	1.10	1.02–1.33	0.038
WBC (per one unit)	1.31	1.05–1.65	0.025	1.09	1.00–1.38	0.093
IL-6 (per one unit)	1.40	1.15–1.59	< 0.001	1.12	1.02–1.26	0.013
MIF (per one unit)	1.12	1.07–1.18	< 0.001	1.06	1.01–1.15	0.009

^ξ^ Adjusted for age, cavitary lesion, pleural effusion, drug-resistant, disseminated, ALB, CRP, WBC, IL-6 and MIF

*CRP* C-reactive protein, *IL-6* Interleukin 6, *WBC* white blood cell count, *FBG* fasting blood glucose, *MIF* macrophage migration inhibitory factor, *ALB* Albumin

**Table 3:** Logistic regression model for plasma concentrations of MIF using mortality as the dependent variables

MIF quartiles, ng/ml	Death/all patients (%)	Crude OR (95% CI); P^‡^	Multivariable-adjusted; P^ξ‡^
Quartile 1(< 19.1)	3/73 (4.1)	Reference	Reference
Quartile 2 (19.1–23.0)	5/74 (6.8)	1.69 (0.39–7.35); 0.48	1.22 (0.84–4.22); 0.73
Quartile 3 (23.1–28.1)	11/69 (15.9)	4.43 (1.18–16.62); 0.018	2.58 (0.89–8.16); 0.19
Quartile 4(> 28.1)	17/71 (23.9)	7.35 (2.05–26.36); 0.001	3.36 (1.21–15.14); 0.012

^ξ^Adjusted for age, cavitary lesion, pleural effusion, drug-resistant, disseminated, ALB, CRP, WBC, IL-6 and MIF

^‡^*P* value for the trend < 0.001

*OR* odds ratio, *CI* confidence interval, *CRP* C-reactive protein, *IL-6* Interleukin 6, *WBC* white blood cell count, *FBG* fasting blood glucose, *MIF* macrophage migration inhibitory factor, *ALB* Albumin

**Fig. 3:**
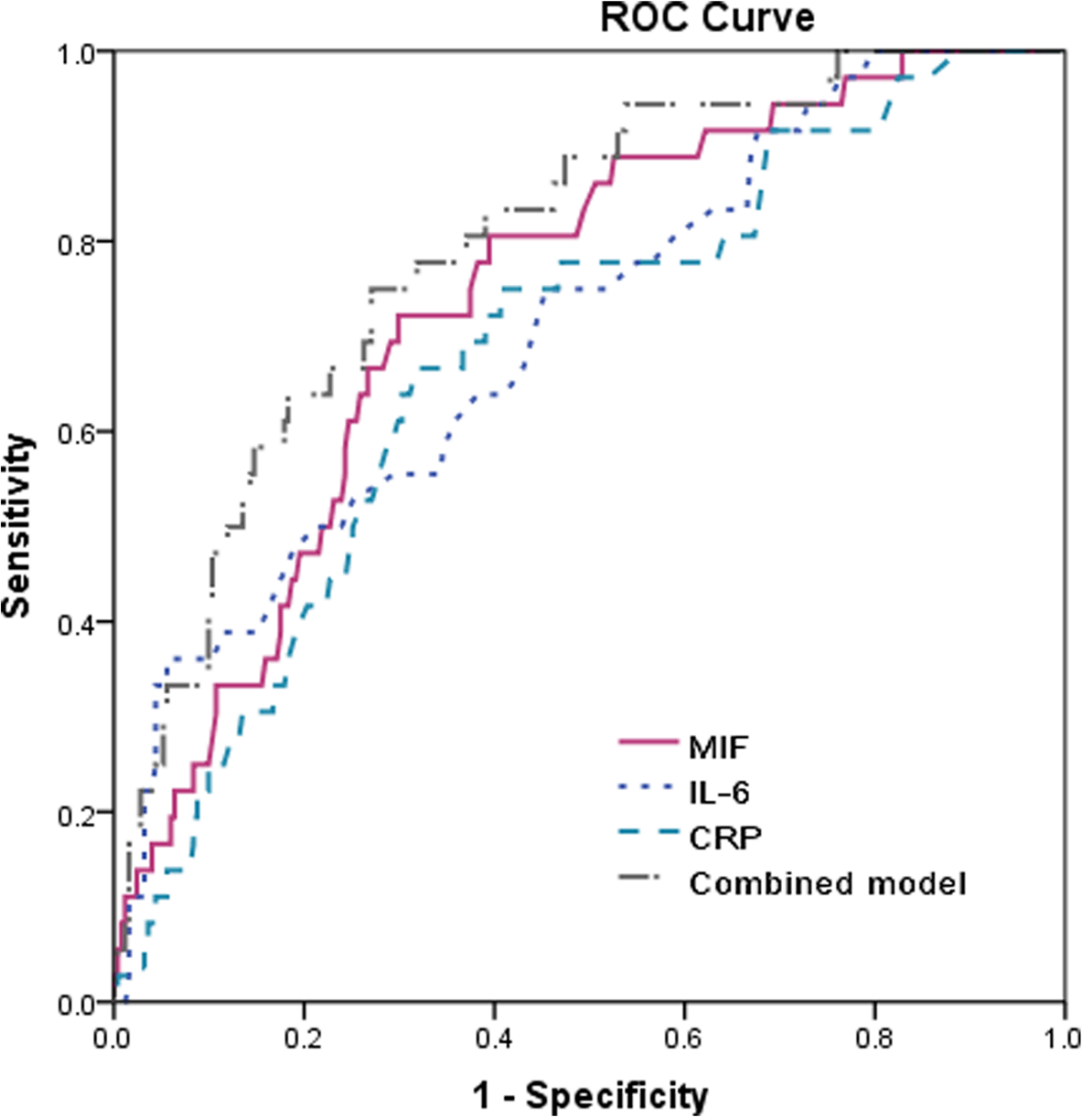
Receiver operator characteristic curve demonstrating sensitivity as a function of 1-specificity for predicting the mortality based on the different biomarkers. Combined model included MIF/CRP/IL-6. CRP=C-reactive protein; IL-6 = Interleukin 6; MIF = Macrophage migration inhibitory factor

The ROC curves were performed to obtain the optimal MIF level. When MIF level was at 25.6 ng/ml, the mortality could be predicted with the highest sensitivity of 72.2% and highest specificity of 70.1% [AUC = 0.74, 95% CI: 0.66–0.82; *P* < 0.001]. The accuracy of MIF level for predicting mortality was higher than that of CRP level [AUC = 0.68, 95% CI: 0.59–0.77, *P* = 0.009], WBC [AUC = 0.59, 95% CI: 0.51–0.68), *P* < 0.001] and IL-6 [AUC = 0.71, 95% CI: 0.62–0.80), *P* = 0.042]. The combination of MIF, CRP and IL-6 provided a better discriminatory accuracy (AUC = 0.80; 95% CI: 0.72–0.87) compared to that of single MIF (*P* < 0.001, Fig. 3). The model was re-analysis after combing MIF with currently established risk indicators. The obtained AUROC (±standard error) was elevated from 0.81 (±0.035) to 0.84 (±0.031), with a significant difference before and after adding the MIF (difference, 0.03[0.004]; *P* = 0.031). The NRI and IDI statistics suggested that the discriminatory classification of died patients and survivors could be significantly increased after combing MIF with established risk indicators (Table 4).

**Table 4:** Serum level of MIF concentrations at admission prediction of Mortality with AUROC

Death	AUROC					
	MIF	Risk factors^a^	Risk factors with MIF	Incremental area (P)^b^	NRI(P)	IDI(P)
At admission	0.74	0.81	0.84	0.03 (0.03)	0.096 (0.008)	0.038 (0.04)

^a^Established risk factors including: age, cavitary lesion, pleural effusion, drug-resistant, disseminated, ALB, CRP, WBC and IL-6

^b^Comparison of AUROCs: established risk factors without MIF levels vs. established risk factors with MIF levels

**Fig. 4:**
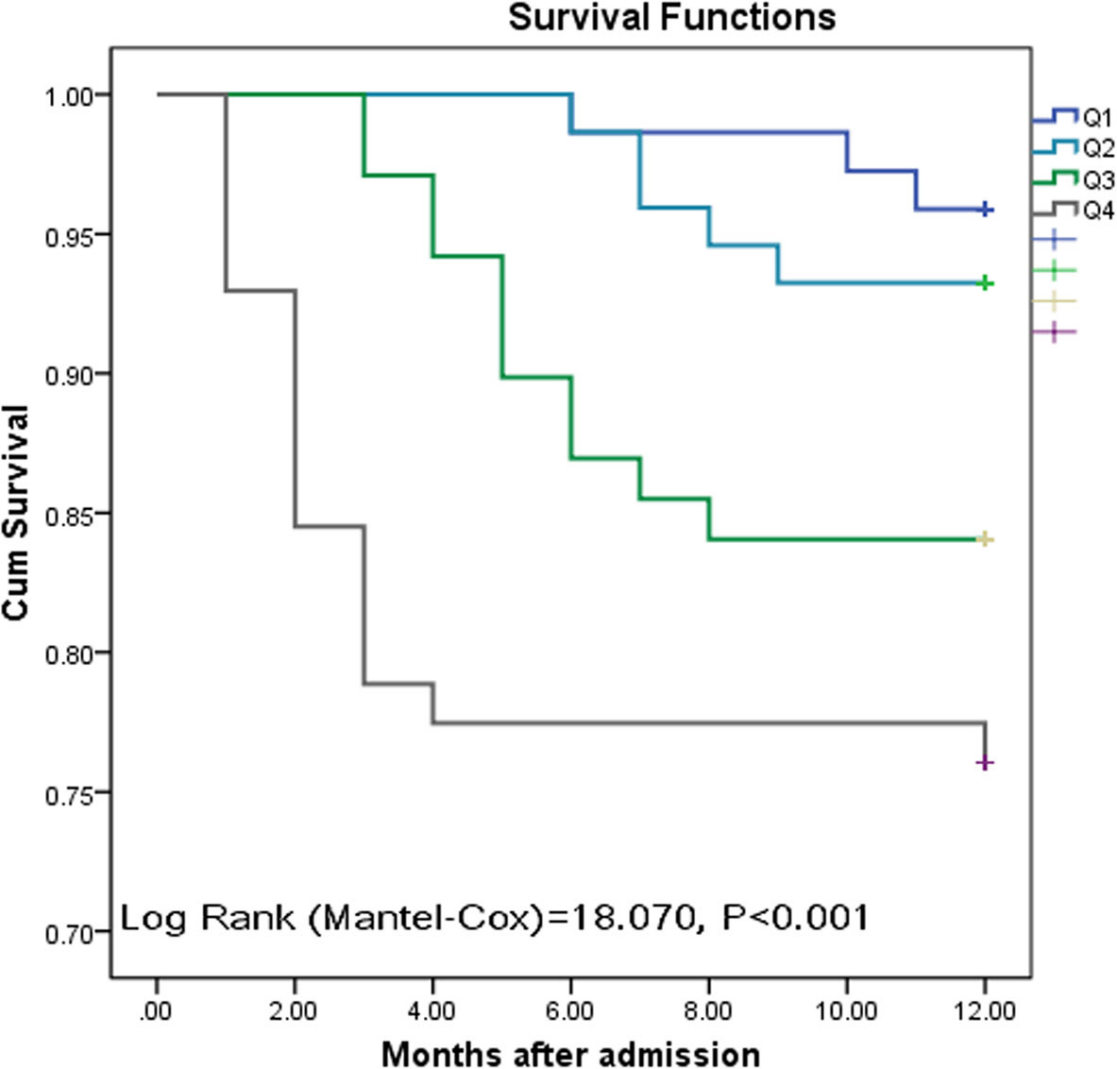
Kaplan-Meier curves analysis for serum MIF concentrations according to quartiles to predict the end point of mortality in PTB patients. Patients in the lowest and second quartiles had a minimal risk for death, in contrast with patients with FABP concentrations in the 3rd and 4th quartiles (*P* < 0.0001). MIF = Macrophage migration inhibitory factor; PTB=Pulmonary Tuberculosis

Kaplan-Meier analyses of all-cause mortality were performed for the four quartiles of MIF levels (Fig. 4). The results indicated that the mortality was related to MIF levels, which was 4.1% in lowest quartile (group 1) and 23.9% in highest quartile (group 4), respectively. The risk of mortality was minimal in patients belonging to the lowest and second quartiles of MIF levels, while it was higher in patients of the third and highest quartiles (*P* < 0.0001).

### The prognostic value of MIF in the validation cohort

The median serum level of MIF in the validation cohort (*N* = 104) was 23.4 ng/ml (IQR, 18.9–28.3) and it was similar compared (*P* = 0.36) to that of in training cohort, which was 23.0 ng/ml (IQR: 19.1–28.1). In addition, these 104 patients were similar in terms of baseline characteristics [age (*P* = 0.48), sex (*P* = 0.86) and BMI (*P* = 0.32)] compared to the training cohort.

At admission, 21 patients (20.2%) had an advanced disease, which was similar compared to the training cohort(*P* = 0.82). The univariate and multivariate logistic regression analysis suggested that when serum level of MIF was increased by 1 ng/ml, the unadjusted and adjusted risk for advanced disease would be elevated by 10% (OR: 1.10 [95% CI: 1.04–1.15], *P* < 0.001) and 5% (OR: 1.05 [95% CI: 1.01–1.10], *P* = 0.004), respectively. Interestingly, disseminated TB was diagnosed in 13 (12.5%) patients, which was similar compared to the training cohort(*P* = 0.64). When serum level of MIF was increased by 1 ng/ml, the unadjusted and adjusted risk for disseminated TB would be increased by 9% (OR: 1.09 [95% CI: 1.03–1.15], *P* = 0.003) and 4% (OR: 1.04 [95% CI: 1.00–1.11], *P* = 0.012), respectively.

In the follow-up, 15 patients died (14.4%), which was similar compared to the training cohort(*P* = 0.63). MIF levels in patients with the outcome of death were also higher than those survived [28.4 ng/ml (IQR: 24.5–33.5) vs. 22.2 ng/ml (IQR: 18.5–26.3); *P* < 0.001]. When serum level of MIF was increased by 1 ng/ml, the unadjusted and adjusted risk for mortality would be increased by 13% (OR: 1.13 [95% CI: 1.08–1.19], *P* < 0.001) and 6% (OR: 1.06 [95% CI: 1.01–1.13], *P* = 0.006), respectively.

With an AUC of 0.75(95% CI: 0.66–0.83), MIF showed a significantly greater discriminatory ability to predict mortality as compared with CRP (AUC, 0.68; 95% CI, 0.60–0.76; *P* < 0.001), WBC (AUC, 0.58; 95% CI, 0.50–0.67; *P* < 0.001) and IL-6 (AUC, 0.71; 95% CI, 0.63–0.78; *P* = 0.035). The combination of MIF, CRP and IL-6 provided a better discriminatory accuracy (AUC = 0.82; 95% CI: 0.74–0.88) compared to that of those biomarkers alone(*P* < 0.05). The model was re-analysis after combing MIF with currently established risk indicators. The obtained AUROC (±standard error) was elevated from 0.81 (±0.033) to 0.85 (±0.028), with a significant difference before and after adding the MIF (difference, 0.04[0.005]; *P* = 0.026). The inclusion of MIF in the prediction model of established risk factors for the prediction of mortality, enhanced the NRI (*P* = 0.006) and IDI (*P* = 0.037) values, confirming the effective reclassification and discrimination.

## Discussion

MTB has been the infectious agent lead to life-threatening human TB [22]. MTB infection was hard to be inhibited since the proinflammatory response could be generally suppressed with the excessive generation of anti-inflammatory cytokines [23]. MIF was a critical cytokine responsible for the innate immunity and inflammatory responses, which was reported to make critical effects in both PTB development [24] and severity [14]. Until now, our study has been the first report to measure serum levels of MIF in PTB and further assess its association with the 12-month prognosis. Our main findings were the following: (1) MIF levels in serum were higher in PTB cases than those of in normal controls; (2) high MIF levels were associated with advanced disease, disseminated and drug-resistant TB; (3) high levels of MIF at admission were related to the elevated risk of mortality in the following 12 months, thus it may be available in predicting the risk of TB associated death; (4) those results have been proven by validated group.

Several cytokines had been secreted by the host immune cells to defense against MTB infection [25], which made effects on immune response in different progressions of TB [26]. Some critical cytokines were secreted in the effective phases of the immune response, such as IFN-γ, TNF-α, and MIF [27]. Then, macrophages could be activated with the microbicidal mechanisms (such as autophagy) of these cytokines [28], which would be involved in MTB suppression [29].

In this study, we found that MIF level was significantly decreased (*P* < 0.01) in 0, 3 and 6 months after treatment, then it entered into a plateau in 9 to 12 months. Similarly, another study reported decreased MIF levels in 2, 4, and 6 months after treatment (*P* < 0.01) [14]. Kuai et al. [15] found that TB could be distinguished from other inflammatory phenotype with profiles of serum MIF, IFN-γ and TNF-α, which might make effects in pathogenesis and be available as indicators of active tuberculosis. In our study, serum level of MIF was also related to the pathogenesis of PTB. Many indicators were reported to be associated with the prognosis of TB patients. Malnutrition was related to elevated risk of mortality in military TB cases [30]. Moreover, serum albumin level was also intense, independent and negative indicator for fatality in TB cases [31]. Ugajin et al. [32] suggested that higher levels of procalcitonin (PCT), a systemic inflammatory protein, could improve the clinical prediction of disseminated tuberculosis and poor prognostic outcomes. Similarly, in this study, we found that low albumin and high MIF serum levels were independently associated with death in PTB patients.

In several previous reports, CRP levels indicated high and positive association with TB, which could be independent of HIV status [33]. IL-6 levels in serum was proposed as another biomarker for indicating the therapeutic effects for PTB [34]. Another study proposed that profiles of dynamic variation of IL-6, IL-15 and IFN-c would assist in TB diagnosis and therapy [35]. In line with those findings, we also found that CRP and IL-6 were associated with TB patients’ mortality.

One study suggested that generally down-regulated expression of MIF alleles could be related to elevated risk of TB [36]. Consistently, previous studies suggested that the PTB risk may be also elevated with − 173 G/C polymorphism of MIF [37, 38]. However, this study did not report any information on gene expression of MIF. While, the relationship between MIF gene expression and MIF levels, as well as the related death in Chinese PTB patients should also be explored in further studies. Furthermore, there were also some other limitations in this study. Firstly, it was worth noting that, the actual MIF levels at lesions may not be accurately reflected with the serum levels of MIF, which was quantified with ELISA. Secondly, the levels of cytokines would be affected with many factors associated with the TB severity, such as symptoms, PTB course, and microorganic status of sputum. Thirdly, the cutoff value of MIF in this study may not be available in other works, since the MIF quantification method has not been standardized. Fourthly, some populations were excluded in this study, such as drug-susceptible TB and HIV-positive cases. Therefore, further study should be performed on these populations to explore the prognostic value of MIF. Finally, the mortality outcome in this study was estimated with all-cause mortality. The mortality causes (TB or non-TB) were not clarified. Thus, the predictive value of MIF in TB and non-TB mortality could not be determined. Further research in the future needs to make this question clear.

## Conclusion

In conclusion, serum level of MIF was a superior indicator for predicting death in PTB patients with negative HIV status, compared to that of CRP or IL-6. In addition, it was significant to realize that the increased serum levels of MIF were associated with disseminated TB and higher risk of fatality.

## Abbreviations

ALB: Albumin
APTB: Active pulmonary tuberculosis
AUC: Area under the curve (AUC) w
BMI: Body mass index
CAP: Community-acquired pneumonia
CI: Confidence interval
CRP: C-reactive protein
CV: Coefficients of variation
FBG: Fasting blood glucose
IDI: Integrated discrimination improvement
IL-6: Interleukin 6
MDR: Multidrug-resistant
MIF: Macrophage migration inhibitory factor
MTB: *Mycobacterium tuberculosis*
NLR: Neutrophil to lymphocyte ratio
NRI: Net reclassification improvement
OR: Odds ratio
PCT: Procalcitonin
PTB: Pulmonary tuberculosis
RDW: Red cell distribution width
ROC: Receiver operating characteristic
TNF-α: Tumor necrosis factor-α
WBC: White blood cell count
